# Glucose negatively affects Nrf2/SKN-1-mediated innate immunity in *C. elegans*

**DOI:** 10.18632/aging.101610

**Published:** 2018-11-15

**Authors:** Le Li, Yi Chen, Changchi Chenzhao, Shuxiang Fu, Qumiao Xu, Jinfeng Zhao

**Affiliations:** 1 Key Laboratory of Nanobiological Technology of Chinese Ministry of Health, Xiangya Hospital ,Central South University, 410008 Changsha, China; 2 Department of Laboratory Medicine, Xiangya Medical School, Central South University, Changsha, 410013 Changsha, China; 3 Xinxiang Medical University, Hongqi District, 453003 Xinxiang, China; 4 Forevertek Biotechnology Co.,Ltd, Building M0, Oversea Graduate Park National High-tech Industrial Zone, 10003 Changsha, China; 5 BGI-Shenzhen, BeiShan Industrial Zone, Yantian District, Shenzhen, Guangdong 518083, China; * Equal contribution

**Keywords:** aging, glucose metabolism, hyperglycemia, innate immunity, Nrf2

## Abstract

High glucose levels negatively affect immune response. However, the underlying mechanisms are not well understood. Upon infection, the round worm *C. elegans* induces multiple gene transcription programs, including the Nrf2/SKN-1-mediated detoxification program, to activate the innate immunity. In this study, we find that high glucose conditions inhibit the SKN-1-mediated immune response to *Salmonella typhimurium*, exacerbate the infection and greatly decrease survival. The effect of glucose shows specificity to SKN-1 pathway, as UPR^mit^ and UPR^ER^ that are known to be induced by infection, are not affected. Hyper-activation of SKN-1 by *wdr-23* RNAi restores partly the immune response and increases the survival rate in response to *S. typhimurium*. In all, our study reveals a molecular pathway responsible for glucose’s negative effect on innate immunity, which could help to better understand diseases associated with hyperglycemia.

## Introduction

One of the hallmarks of diabetic complication is impaired wound healing [1]. This probably is resulted from a negative effect of high glucose on immune response. It is shown that high glucose levels can inhibit the normal function including chemotaxis, phagocytosis, killing of polymorphonuclear cells such as monocytes and macrophages [2]. Consistently, in *C. elegans*, high glucose can cause rapid aging and greatly shorten the lifespan [3-5]. As pathogenic infections also strongly decrease the lifespan of *C. elegans* [6,7], glucose may speed up aging by compromising the immune response. However, whether this is the case and what are the underlying molecular mechanisms remain poorly understood.

Previously, efforts have been directed to delineate the signaling pathways that mediate the glucose signaling in infection-related diseases. It was found in mice that several key molecular pathways participate in the pathogenic process, such as the protein kinase C pathway, the protein glycation pathway, TNF-α pathway, etc [8]. These pathways are interconnected and dynamically regulate immunity in response to high glucose. Deregulation of these pathways tends to elevate the intracellular levels of reactive oxygen species (ROS), increase oxidative damage and cause abnormal protein modification, which could contribute to the disease state of diabetes [8-10]. Therefore, anti-oxidation pathways could play an important role in the regulation of glucose suppression of immune system.

One of the major anti-oxidation pathway is controlled by the nuclear factor erythroid 2 (NFE2)-related factor 2 (Nrf2) [11,12]. Nrf2 is a member of the cap 'n' collar (CNC) subfamily of basic region leucine zipper (bZip) transcription factors. Nrf2 protein is highly conserved and its *C. elegans* homolog SKN-1 functions in similar way to bind promoters of oxidative stress-related genes [12]. The regulation of both Nrf2 and SKN-1 is largely through posttranslational modifications and proteasome-mediated degradation. Under normal conditions, Nrf2 is sequestered by its associated protein KEAP-1 in the cytoplasm, targeted for ubiquitination and proteasome-mediated degradation [13]. Upon stress, posttranslational modifications of either Nrf2 or KEAP-1 cause release of the Nrf2 into the nucleus, which then drives transcription of stress responsive genes. Nrf2 has been implicated in many diseases [14,15] and also the aging process [12,16]. Similarly in *C. elegans*, SKN-1 is also targeted by proteasome for degradation upon stress conditions. However, as *C. elegans* lacks KEAP-1 ortholog [12], WDR-23 has been proposed to be play similar roles in preventing Nrf2 degradation [17].

Aging modulators are commonly targeted by pathogen defense pathways. For example, in *C. elegans*, SKN-1 is required for many lifespan extension events [12], and it is also an essential regulator of innate immunity [18]. The well-known aging regulator DAF-16 is required for protection against strains that kill *C. elegans* slowly by gut colonization [19,20]. Recently, DAF-16 is also found to be phosphorylated by MBK-1 [21], a DYRK kinase that promotes resistance to P. aeruginosa [22]. In mammals, several studies suggest Nrf2 to be an important modulator of innate immunity. Loss of Nrf2 in mice renders the animal vulnerable to infection by viruses [23]. Similarly, disruption of Nrf2 dramatically increases susceptibility to cecal ligation and puncture-induced sepsis [24]. On the other hand, activation of Nrf2 reduces the infection by *Salmonella typhimurium* [25]. In addition, mice fed with sulforaphane, a pharmacologic activator of Nrf2, show significant resistance to infection by bacteria such as *Pseudomonas aeruginosa* and *Plasmodium. falciparum* [26,27]. All these results suggest an important role of Nrf-2/SKN-1 in fighting against infectious disease. However, whether Nrf2/SKN-1 plays a role in high glucose-induced immunity malfunction remains unknown.

We are interested to know the functions of SKN-1 in high glucose conditions. As the physiology of the round worm *C. elegans* is severely affected by glucose, this animal model is especially suited to study glucose toxicity. In this report, we examine the response of SKN-1 pathway to *S. typhimurium* in the presence or absence of glucose and found that glucose dampens SKN-1 activity, increases infection and limits survival. Activation of SKN-1 by knocking down *wdr-23* expression alleviates the negative effect of glucose on infection and lifespan of *C. elegans*. This study may help to better understand the pathological pathways underlying human diseases related to high glucose conditions.

## RESULTS

### Glucose renders *C. elegans* vulnerable to infection by *S. typhimurium*

**Figure 1:**
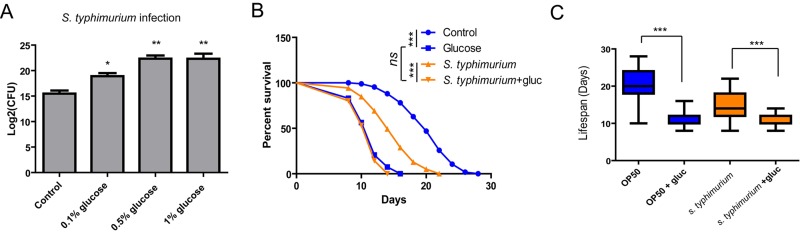
**Glucose medium exacerbates *S. typhimurium* infection and shortens survival.** (**A**) Glucose increases *S. typhimurium* infection in *C. elegans*. Animals cultured in the presence and absence of various concentrations of glucose were infected with *S. typhimurium* at L4 or young adult stage for 2 days. The numbers of infected pathogen were determined by lysing 20 worms and colony forming assay of live *S. typhimurium* inside the worms. Colony forming unit (CFU) was plotted using Log2. Data from two independent experiments were pooled and plotted. Error Bars stands for standard error of the mean (SEM). P values were obtained by student’s t-test. *, P<0.01, **, P<0.001. (**B**) Glucose decreases lifespan of infected animals. Lifespan and infection were carried out at 20 ºC. Animals were infected with *S. typhimurium* at L4 or young adult stage for 2 days then transferred to normal NGM plates. Survival of control and infected animals were recorded every other day. Data were collected from two independent experiments with number of worms >100. See Table S1 for details. (**C**) Comparison of killing effect in the presence and absence of glucose. Lifespan of animals (n>100) were plotted in Whiskers box. P values were obtained by Log-rank test. ***, P<0.0001.

To test if glucose would affect the innate immunity in *C. elegans*, we infected the animals with *Salmonella typhimurium* (*S. typhimurium*) by exposing L4 stage worms to the pathogen for 48 hours. The number of live bacteria inside the worms is commonly used to evaluate the extent of infection in *C. elegans* [28]. To determine the degree of infection, we washed day-5 adult worms extensively to get rid of the bacteria on the outer cuticle. We then lysed the worms and plate the lysate in order to count the number of live bacteria by colony-forming assay. As shown in Fig. 1A, glucose supplemented in culture medium already robustly increased the infection, with 0.5% reaching the highest. We therefore used 0.5% of glucose for the rest experiments in this study if not otherwise stated.

As infection is highly associated with survival rate, we determined if glucose would affect survival of animals after infection by pathogen *S. typhimurium*. After infection, we transferred the animals to normal NGM plate with non-pathogenic OP-50 bacteria and recorded survival every other day until the worms were all dead. Consistent with the enhanced infection in Fig. 1A, glucose supplementation decreased survival rate of worms infected by *S. typhimurium*. Glucose also shortened the lifespan of *C. elegans* in the non-pathogenic bacteria such as OP-50 (Fig. 1B), as has been reported by several studies before [3-5]. Both *S. typhimurium* and glucose shortened lifespan of *C. elegans* in a non-additive manner (Fig. 1C).

### Glucose prevents *S. typhimurium* from activating SKN-1

**Figure 2:**
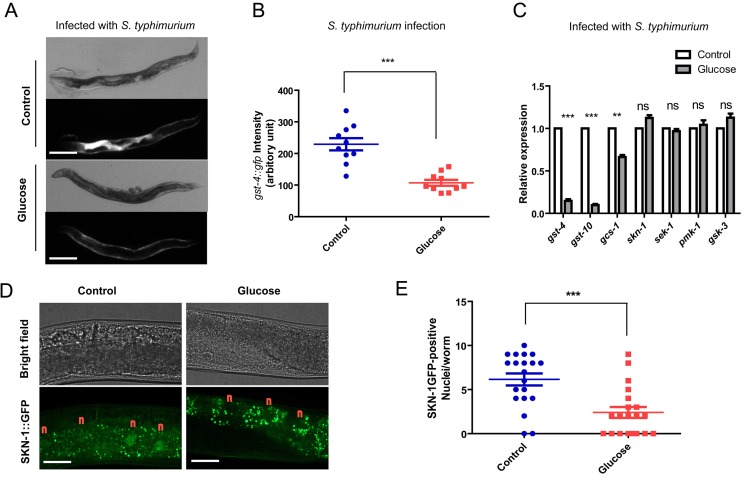
**Glucose medium decreases SKN-1 activity.** (**A**) SKN-1 reporter *gst-4*::*gfp* is suppressed by high glucose medium. Animals expressing the SKN-1 reporter *gst-4*::*gfp* were cultured in medium supplemented with and without 0.5% glucose from L1 stage to L4/young adult stag, then transferred to infection plate without glucose for 2 days before imaging. Shown are representative images of at least 4 independent experiments (20 animals each). Scale bars are 200 µm. (**B**) Quantification of experimental results in Fig 2A by measuring the signal intensity of 10 animals from 1 experiment by ImageJ software. P values were obtained by student’s t-test. ***, P<0.0001. Error bars indicates standard error of the mean (SEM). (**C**) SKN-1 target genes (*gst-4*, *gst-10*, *gcs-1*) but not *skn-1* and upstream kinase genes (*sek-1*, *pmk-1*, *gsk-3*) are affected by glucose. Animals raised on medium with and without glucose from L1 to L4/young adult stage were infected by *S. typhimurium* for 2 days. mRNA were extracted and reverse transcribed to cDNA. Quantitative RT-PCR was conducted using established primer sets and protocols. Shown are representative data from 1 of 2 independent experiments. Error bars indicate standard error of the mean (SEM) of 3 replicates. P values were obtained by student’s t-test. **, P<0.001; ***, P<0.0001; ns, not significant. (**D**) Glucose inhibits SKN-1 nuclear localization upon infection. Transgenic *C. elegans* expressing *skn-1::gfp* were raised on medium with and without glucose from L1 stage to L4/young adult stag, then infected with S. *typhimurium* for 2 days before imaging. Shown are representative image of 2 independent experiments. “n” marks above the nucleus of intestinal cells. Scale bars are 40 µm. The punctate signals in the intestine are non-specific signals as also shown in Fig. S4. (**E**)Quantification of experimental results in Fig 2D by counting the SKN-1::GFP positive nuclei/worms of about 20 worms. Shown are representative data from 1 of the 2 independent experiments. Error bars stands for standard error of the mean (SEM). P values were obtained by student’s t-test. ***, P<0.0001.

There are several transcriptional programs that mediate the innate immune response in *C. elegans*. Next, we wanted to know if SKN-1-mediated transcriptional program would be negatively affected by high glucose conditions. To this end, we first asked if promoter activity of *gst-4* gene, a direct transcriptional target of SKN-1, could be changed by feeding glucose. This is revealed by examining directly GFP expression from the *gst-4* promoter (P*gst-4::gfp*) [29]. *gst-4::gfp* can be activated by many stressors, but in response to pathogen, it was robustly activated in a SKN-1-dependent manner [18]. Therefore, we used *gst-4::gfp* as a proxy for SKN-1 activity in response to pathogen. As a result, infection by *S. typhimurium* robustly increased the *gst-4::gfp* expression, indicating a strong SKN-1 activation (Fig. 2A). However, when raised on NG medium supplemented with glucose, SKN-1 activation was greatly reduced (Fig. 2A and 2B). Consistent with previous studies [18], the P*gst-4::gfp* reporter was not activated by non-pathogenic OP-50 (Fig. S1); adding glucose suppressed *S. typhimurium*-induced GFP, but did not change the GFP levels in OP-50 medium (Fig. S1). We have observed similar results in RNAi experiment using HT115 bacteria (Fig. 4A and 4B). We conclude that the glucose effect we observed is specific to conditions of infection by *S. typhimuri*um.

Second, we tested the expression of several Nrf-2 target genes (*gst-4*, *gcs-1* and *gst-10*) by qRT-PCR. Consistently, induction of these SKN-1 target genes by *S. typhimurium* was suppressed when glucose was presented in the culture medium (Fig. 2C). Without induction by *S. typhimurium* infection, *gst-4* expression was low and not affected by glucose (Fig. S2), consistent with the gst-4::gfp reporter in Fig. S1. Expression of genes encoding *skn-1* and upstream kinase such as *sek-1*, *pmk-1* and *gsk-3* were not affected by glucose (Fig. 2C), suggesting that the mRNA levels of upstream regulators were not affected (Fig. 2C). Third, we examined the subcellular localization of SKN-1/Nrf2 protein under high glucose conditions. Transcription of SKN-1/Nrf2 target genes requires the accumulation of SKN-1/Nrf2 in the nucleus upon infection [18]. We found that glucose strongly attenuated the accumulation of SKN-1::GFP in the intestinal cells of *C. elegans* (Fig. 2D and 2E). Therefore, glucose targets SKN-1/Nrf2 to impair immune response to *S. typhimurium* in *C. elegans*.

### Glucose specifically inhibits SKN-1-mediated immune response to *S. typhimurium*

**Figure 3:**
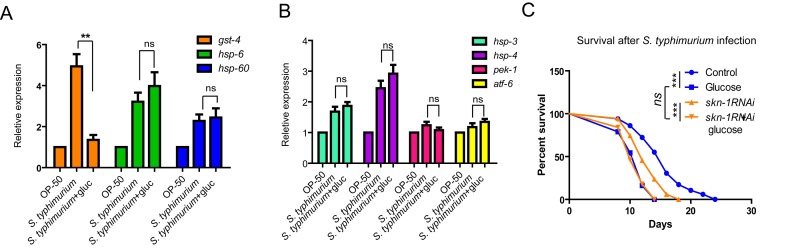
**Glucose does not affect expression of marker genes of UPR^mt^ or UPR^ER^.** (**A**) SKN-1 target gene (*gst-4*) but not marker genes of mitochondrial unfolded protein response (UPR^mt^) were affected by glucose. Animals raised on medium with and without glucose from L1 to L4/young adult stage were infected by *S. typhimurium* for 2 days. mRNA were extracted and reverse transcribed to cDNA. Quantitative RT-PCR was conducted using established primer sets (Table S4). P values were obtained by student’s t-test. **, P<0.001; ns, not significant. (**B**) Genes known to be induced by endoplasmic reticulum unfolded protein response (UPR^ER^) were not affected by glucose. Animals raised on medium with and without glucose from L1 to L4/young adult stage were infected by *S. typhimurium* for 2 days. mRNA were extracted and reverse transcribed to cDNA. Quantitative RT-PCR was conducted using established primer sets (Table S4). Two independent experiments shows similar results and one of them are shown. P values were obtained by student’s t-test. ns, not significant. (**C**) Glucose and *skn-1* RNAi knockdown is not additive in decreasing *C. elegans*’ lifespan. Lifespan and infection were carried out at 20 ºC. Animals raised on medium with and without glucose from L1 to L4/young adult stage were infected by *S. typhimurium* for 2 days, then transferred back to non-infected OP-50 bacteria plate for the rest of life. Survival were recorded every other day until all died. Data were collected from two independent experiments with number of worms >100. See Table S2 for details.

We also tested if other pathogen defense pathways were affected by glucose. The mitochondrial unfolded protein response (UPR^mit^) and the endoplasmic reticulum unfolded protein response (UPR^ER^) are key stress responses that are also induced upon certain pathogen infection [30,31]. We asked if *S. typhimurium* could induce similar responses and how they were affected by glucose. To this end, we examined several downstream genes in these two pathways by qRT-PCR. The expression of *hsp-6* and *hsp-60* genes are well established markers for UPR^mit^ [32], while *hsp-3*, *hsp-4*, *pek-1* and *atf-6* are key genes involved in UPR^ER^ [33]. We found that *hsp-6*, *hsp-60*, *hsp-3* and *hsp-4* were significantly increased in expression upon infection by *S. typhimurium*. However, for all these genes, we detected no significant difference between worms cultured with and without glucose (Fig. 3A and 3B). These data suggest that the glucose specifically targets SKN-1 pathway to affect immune response in *C. elegans*.

Loss of SKN-1 shortens lifespan. Glucose can also greatly shorten lifespan. If glucose shortens lifespan through inhibiting SKN-1, one would expect that, knocking down *skn-1* expression can no longer shorten lifespan in the presence of glucose. Indeed, we confirmed that wild-type N2 worms cultured with SKN-1 RNAi bacteria were much shorter lived than the control. However, when the worms were cultured on the same RNAi bacteria plate supplemented with 0.5% of glucose, lifespan was no longer shortened (Fig. 3C).

### SKN-1 hyper-activation diminishes the negative effect of glucose on immunity

**Figure 4:**
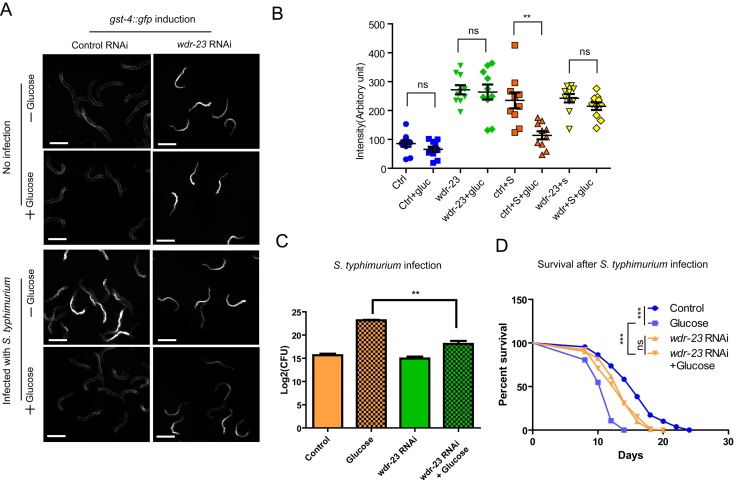
Activation of SKN-1 pathway mitigates the negative effect of glucose on immune response to *S. typhimurium*. (**A**) Knocking down *wdr-23* bypasses glucose to activate *gst-4::gfp*. Animals expressing the SKN-1 reporter *gst-4*::*gfp* were fed bacteria expressing double-stranded RNA of *wdr-23* from L1 stage to L4/young adult stage on medium with and without glucose, then infected with S. *typhimurium* for 2 days before imaging. Two independent trials gave similar results and data from one of them were shown. Scale bars are 600 µm. (**B**) Quantifications of *gst-4*::*gfp* intensity of 10 animals in images shown in A by ImageJ software. Ctrl, control; gluc, 0.5% glucose; S, S. typhimurium. P values were obtained by student’s t-test. **, P<0.001. ns, not significant. (**C**) Knocking down *wdr-23* alleviates glucose’s negative effect on infection. Animals fed bacteria expressing double-stranded RNA of *wdr-23* from L1 stage to L4/young adult stage on medium with and without glucose were infected with S. *typhimurium* for 2 days. The numbers of infected pathogen were determined colony forming assay of live S. typhimurium inside the worms. Colony forming unit (CFU) was plotted using Log2. P values were obtained by student’s t-test. **, P<0.001. (**D**) Knocking down *wdr-23* prevents glucose from shortening lifespan of infected *C. elegans*. Lifespan and infection were carried out at 20 ºC. Animals fed bacteria expressing double-stranded RNA of *wdr-23* from L1 stage to L4/young adult stage on medium with and without glucose were infected with S. *typhimurium* for 2 days. Worms were then transferred to non-infected RNAi bacterial plates. Survival was recorded every other day until all worms died. Data were collected from two independent experiments with number of worms >100. See Table S3 for details.

Since glucose suppress the innate immunity through inhibiting SKN-1, it is possible that hyper-activation of SKN-1 could reverse the negative effect of glucose. To test this idea, we activated the SKN-1 activity through RNAi knocking down the expression of *wdr-23* gene. WDR-23 is analog of mammalian KEAP-1, which binds directly to SKN-1 to promote its proteasome-mediated degradation [34]. Knocking down *wdr-23* can accumulate SKN-1 rapidly in the nucleus and activate transcription of SKN-1 target genes such as *gst-4* [17]. Confirming the previous results, we found that the promoter activity of *gst-4* was highly induced by *wdr-23* RNAi knockdowns, as revealed by *gst-4::gfp* reporter (Fig. 4A and 4B). Interestingly, when *wdr-23* was knocked down, *gst-4::gfp* expression was no longer affected by glucose (Fig. 4A), suggesting that WDR-23 is functioning in the downstream or in parallel to glucose signaling.

We then evaluated the biological significance of *wdr-23* knockdown by examining directly the infection degree through colony assay. Our results showed that the negative effect of glucose on infection was significantly mitigated by *wdr-23* knockdowns (Fig. 4B). Furthermore, upon *wdr-23* knockdowns, glucose’s negative effect on survival in the presence of *S. typhimurium* was prevented (Fig. 4C and Fig. S3), suggesting that forced activation of SKN-1 could be sufficient to reverse hyperglycemia -induced infection. In the absence of *Salmonella. typhimurium*, however, *wdr-23* RNAi showed no interaction with glucose (Fig. S3). These results suggest that glucose enhances the pathogen killing effect through WDR-23.

## DISCUSSION

It has been known for a long time that high glucose condition can exacerbate pathogenic infection in human [2,8]. But the underlying mechanism remains incompletely understood. By using the round worm *C. elegans* as a model, we aimed to better understand the mechanisms by which glucose modulates the innate immune response. Our studies reveal SKN-1 as a key mediator of glucose’s negative effect on infection by *Salmonella typhimurium*. SKN-1 is homologous to human Nrf2, which has been implicated in many human diseases such as cancers, neurodegenerative disease and diabetic Nephropathy [35-37]. Our study therefore may have significant relevance to the human physiology and pathology.

Recent efforts to understand the underlying mechanisms that lead to compromised immune response by hyperglycemia have implicated several pathways, such as PKC, ROS and polyol pathway [8]. It is interesting to note that dysregulation of these pathways are prone to increase intracellular ROS levels [8,9]. As ROS can activate Nrf2/SKN-1, our finding of SKN-1 in mediating glucose’s effect on immunity is in line with abovementioned results [8,9]. Interestingly, because high glucose metabolism is generally known to accelerate the production of ROS, one would expect that SKN-1 would be activated, instead of suppressed by glucose as shown in this study. One possibility for the increased ROS in glucose metabolism is that it is a result of SKN-1 inhibition, which will be in line with the data presented. Another possible explanation could be that the prominent mechanism for glucose to suppress Nrf2/SKN-1 is not mediated by ROS, but through other unknown molecules. One can imagine that there could be many possible mechanisms that target transcription, translation and posttranslational modification of certain components in the Nrf2/SKN-1 pathway. Gaining insight into the underlying mechanism requires further efforts and expertise from multiple fields of study.

Another very interesting possibility that deserves special attention is that high glucose condition may suppress the generation of ROS, which then prevent the activation of SKN-1. Nrf2/SKN-1 is a key regulator of the innate immunity [18,24]. It is known that lipopolysaccharides from the invading microbes trigger a pathway that lead to the generation of superoxide. The superoxide then disrupts the function of KEAP-1/WDR-23, a negative regulator of Nrf2/SKN-1 [38,39], therefore activating Nrf2/SKN-1. High glucose may act on these molecules in the upstream of KEAP-1/WDR-23. This is consistent with our result that by RNAi knocking down of *wdr-23*, glucose’s negative effect on SKN-1 target genes expression and survival upon infection is alleviated. Recently, several groups found that ROS, at low levels, can serve as signal to activate protective programs in order to counteract further accumulation of deleterious ROS [29,40]. It is found that high glucose can suppress the increase of such ROS [4], negatively affecting key biological processes that determine survival. Similar mechanisms could exercise in the interface of glucose and infection, resulting in inhibition of innate immunity in *C. elegans* upon *S. typhimurium* infection. Our results may suggest a conserved and novel pathway underlying the negative effect of hyperglycemia on human immune response. It will be of great interest to examine in other animal models, especially mammalian model such as mice to see if the current finding is conserved in regulation of hyperglycemia-mediated diseases, such as diabetes.

Interestingly, although opposing in terms of *gst-4::gfp* induction, both *S. typhimurium* and glucose shortened lifespan of *C. elegans* in a non-additive manner (Fig. 1C). Consistent with our study, another recent report also shows that glucose has no additive effect on killing, sometimes even extends the survival of *C. elegans* that are infected with certain pathogens [41]. These results suggested that *S. typhimurium* and glucose may shorten lifespan through a common pathway. Alternatively, as lifespan/survival is a downstream phenotype that can be complicated by many factors, another possibility could be that the lifespan is affected by other pathways such as mitochondrial and/or ER unfolded protein responses, which are known to contribute to lifespan extension in *C. elegans* [30,42].

## MATERIALS AND METHODS

### Strains and medium

Transgenic reporter strains were CL2166 (*dvIs19* [pAF15 (*gst-4::*GFP::NLS)] III) and LD1008 (ldEx9 [skn-1(operon)::GFP + rol-6(su1006)]), which were crossed to the control strain (N2 Bristol wild-type) at least 3 times. Standard nematode growth medium (NGM) were prepared according to Wormbook (http://www.wormbook.org/chapters/www_strainmaintain/strainmaintain.html). All *C. elegans* strains were maintained at 20 ˚C on standard NGM plates seeded with OP-50 bacteria at least 3 generations before experiments. For glucose medium, glucose was prepared in 30% (w/v) stock solution and autoclaved. Stock glucose solution was added to autoclaved NG medium at final concentration of 0.1%, 0.5% and 1%, before pouring the plates.

### Pathogen infection and survival measurement

*S. typhimurium* infections were done as described in [28]. Briefly, pathogenic bacteria were cultured overnight and plated on NGM agar plates overnight. L4 and young adult worms were transferred to *S. typhimurium*-containing plate without glucose and raised for 48 hours, then transferred back to plates seeded with non-pathogenic OP-50 or HT115(DE3) when doing RNAi knockdown. To determine the degree of infection, 20 day-5 worms were washed extensively then mechanically disrupted by homogenizer in 0.5 ml of M9 buffer. The homogenized solution was serial diluted and plated on *S. typhimurium*-selective XLD agar plates (Xylose Lysine Desoxycholate, EMD Chemical Inc.) to count the titer. To determine survival rate, infected animals were examined for death every other day and percentage of death were plotted using Graphpad Prism 5.

### RNAi treatment

RNAi clones were from a collection initially generated in Julie Ahringer’s laboratory [43], with the same bacteria strain (HT115) containing the empty vector L4440 as control. RNAi experiments was conducted by feeding worms on agar plates with bacteria expressing double-stranded RNA (dsRNA) corresponding to genes to be knocked down. Specifically, bacteria bearing a *wdr-23* DNA fragment were cultured to log phase and seeded on NG plates containing 50 ug/mL Carbenicillin and 1 mM Isopropyl β-D-1-thiogalactopyranoside (IPTG) for at least 24 hours to induce dsRNA expression. L1 stage worms were then transferred to and maintained on the RNAi plate throughout life, gene knockdowns were confirmed by activation of *skn-1* reporter *gst-4::gfp*.

### Quantitative real time polymerase chain reaction (qRT-PCR)

The qRT-PCR was done similarly as described in [44]. Briefly, worms were washed from agar plates with ice-cold M9 buffer, total mRNA were then extracted by Trizol. mRNA was reverse-transcribed to cDNA using QIAGEN One-Step RT-PCR Kit. Quantitative PCR was performed using SYBR Green 2X Mater Mix (Applied Biosystems). Gene expression levels were normalized to actin (*ACT1*) and expressed as fold changes to that of the wild-type. Primers are published before [45], which are also listed in Table S4.

### Microscopic imaging

Worms were paralyzed in 1mM levamisole solution and mounted on 3% of agarose gel pad, covered with cover slide and subject to immediate examination by fluorescent microscope. Worms expressing *gst-4::gfp* were imaged with stereo microscope (Leica Microsystem). Worms expressing SKN-1::GFP were imaged through confocal microscope (Perkin Elmer UltraView Vox Spinning Disk Confocal). Signals from individual animals were quantified with Image J software and plotted as dots.

### Statistical analysis

Survival curves and associated data including mean lifespan, standard errors and P values were generated by bioinformatics software Graphpad Prism. P values for bar data are based on student’s t-test and survival curves are based on log rank test. P < 0.01 was considered statistically significant.

## Supplementary Material

Supplementary Figures

Supplementary Tables

## References

[1] Falanga V. Wound healing and its impairment in the diabetic foot. Lancet. 2005; 366:1736–43. 10.1016/S0140-6736(05)67700-8 16291068

[2] Geerlings SE, Hoepelman AI. Immune dysfunction in patients with diabetes mellitus (DM). FEMS Immunol Med Microbiol. 1999; 26:259–65. 10.1111/j.1574-695X.1999.tb01397.x 10575137

[3] Lee SJ, Murphy CT, Kenyon C. Glucose shortens the life span of C. elegans by downregulating DAF-16/FOXO activity and aquaporin gene expression. Cell Metab. 2009; 10:379–91. 10.1016/j.cmet.2009.10.003 19883616 PMC2887095

[4] Schulz TJ, Zarse K, Voigt A, Urban N, Birringer M, Ristow M. Glucose restriction extends Caenorhabditis elegans life span by inducing mitochondrial respiration and increasing oxidative stress. Cell Metab. 2007; 6:280–93. 10.1016/j.cmet.2007.08.011 17908557

[5] Schlotterer A, Kukudov G, Bozorgmehr F, Hutter H, Du X, Oikonomou D, Ibrahim Y, Pfisterer F, Rabbani N, Thornalley P, Sayed A, Fleming T, Humpert P, et al. C. elegans as model for the study of high glucose- mediated life span reduction. Diabetes. 2009; 58:2450–56. 10.2337/db09-0567 19675139 PMC2768179

[6] Labrousse A, Chauvet S, Couillault C, Kurz CL, Ewbank JJ. Caenorhabditis elegans is a model host for Salmonella typhimurium. Curr Biol. 2000; 10:1543–45. 10.1016/S0960-9822(00)00833-2 11114526

[7] Kurz CL, Tan MW. Regulation of aging and innate immunity in C. elegans. Aging Cell. 2004; 3:185–93. 10.1111/j.1474-9728.2004.00108.x 15268752

[8] Graves DT, Kayal RA. Diabetic complications and dysregulated innate immunity. Front Biosci. 2008; 13:1227–39. 10.2741/2757 17981625 PMC3130196

[9] Giacco F, Brownlee M. Oxidative stress and diabetic complications. Circ Res. 2010; 107:1058–70. 10.1161/CIRCRESAHA.110.223545 21030723 PMC2996922

[10] Araki E, Nishikawa T. Oxidative stress: A cause and therapeutic target of diabetic complications. J Diabetes Investig. 2010; 1:90–96. 10.1111/j.2040-1124.2010.00013.x 24843413 PMC4008021

[11] Suzuki T, Yamamoto M. Stress-sensing mechanisms and the physiological roles of the Keap1-Nrf2 system during cellular stress. J Biol Chem. 2017; 292:16817–24. 10.1074/jbc.R117.800169 28842501 PMC5641889

[12] Blackwell TK, Steinbaugh MJ, Hourihan JM, Ewald CY, Isik M. SKN-1/Nrf, stress responses, and aging in Caenorhabditis elegans. Free Radic Biol Med. 2015; 88:290–301. 10.1016/j.freeradbiomed.2015.06.008 26232625 PMC4809198

[13] Villeneuve NF, Lau A, Zhang DD. Regulation of the Nrf2-Keap1 antioxidant response by the ubiquitin proteasome system: an insight into cullin-ring ubiquitin ligases. Antioxid Redox Signal. 2010; 13:1699–712. 10.1089/ars.2010.3211 20486766 PMC2966484

[14] Syu JP, Chi JT, Kung HN. Nrf2 is the key to chemotherapy resistance in MCF7 breast cancer cells under hypoxia. Oncotarget. 2016; 7:14659–72. 10.18632/oncotarget.7406 26894974 PMC4924742

[15] Rotblat B, Melino G, Knight RA. NRF2 and p53: januses in cancer? Oncotarget. 2012; 3:1272–83. 10.18632/oncotarget.754 23174755 PMC3717791

[16] Dues DJ, Andrews EK, Schaar CE, Bergsma AL, Senchuk MM, Van Raamsdonk JM. Aging causes decreased resistance to multiple stresses and a failure to activate specific stress response pathways. Aging (Albany NY). 2016; 8:777–95. 10.18632/aging.100939 27053445 PMC4925828

[17] Choe KP, Przybysz AJ, Strange K. The WD40 repeat protein WDR-23 functions with the CUL4/DDB1 ubiquitin ligase to regulate nuclear abundance and activity of SKN-1 in Caenorhabditis elegans. Mol Cell Biol. 2009; 29:2704–15. 10.1128/MCB.01811-08 19273594 PMC2682033

[18] Papp D, Csermely P, Sőti C. A role for SKN-1/Nrf in pathogen resistance and immunosenescence in Caenorhabditis elegans. PLoS Pathog. 2012; 8:e1002673. 10.1371/journal.ppat.1002673 22577361 PMC3343120

[19] Garsin DA, Villanueva JM, Begun J, Kim DH, Sifri CD, Calderwood SB, Ruvkun G, Ausubel FM. Long-lived C. elegans daf-2 mutants are resistant to bacterial pathogens. Science. 2003; 300:1921. 10.1126/science.1080147 12817143

[20] Miyata S, Begun J, Troemel ER, Ausubel FM. DAF-16-dependent suppression of immunity during reproduction in Caenorhabditis elegans. Genetics. 2008; 178:903–18. 10.1534/genetics.107.083923 18245330 PMC2248360

[21] Mack HI, Zhang P, Fonslow BR, Yates JR. The protein kinase MBK-1 contributes to lifespan extension in *daf-2* mutant and germline-deficient *Caenorhabditis elegans.* Aging (Albany NY). 2017; 9:1414–32. 10.18632/aging.101244 28562327 PMC5472741

[22] Shao Z, Zhang Y, Ye Q, Saldanha JN, Powell-Coffman JA. C. elegans SWAN-1 Binds to EGL-9 and regulates HIF-1-mediated resistance to the bacterial pathogen Pseudomonas aeruginosa PAO1. PLoS Pathog. 2010; 6:e1001075. 10.1371/journal.ppat.1001075 20865124 PMC2928816

[23] Nagai N, Thimmulappa RK, Cano M, Fujihara M, Izumi-Nagai K, Kong X, Sporn MB, Kensler TW, Biswal S, Handa JT. Nrf2 is a critical modulator of the innate immune response in a model of uveitis. Free Radic Biol Med. 2009; 47:300–06. 10.1016/j.freeradbiomed.2009.04.033 19410644 PMC2700746

[24] Thimmulappa RK, Lee H, Rangasamy T, Reddy SP, Yamamoto M, Kensler TW, Biswal S. Nrf2 is a critical regulator of the innate immune response and survival during experimental sepsis. J Clin Invest. 2006; 116:984–95. 10.1172/JCI25790 16585964 PMC1421348

[25] Nairz M, Schleicher U, Schroll A, Sonnweber T, Theurl I, Ludwiczek S, Talasz H, Brandacher G, Moser PL, Muckenthaler MU, Fang FC, Bogdan C, Weiss G. Nitric oxide-mediated regulation of ferroportin-1 controls macrophage iron homeostasis and immune function in Salmonella infection. J Exp Med. 2013; 210:855–73. 10.1084/jem.20121946 23630227 PMC3646493

[26] Harvey CJ, Thimmulappa RK, Sethi S, Kong X, Yarmus L, Brown RH, Feller-Kopman D, Wise R, Biswal S. Targeting Nrf2 signaling improves bacterial clearance by alveolar macrophages in patients with COPD and in a mouse model. Sci Transl Med. 2011; 3:78ra32. 10.1126/scitranslmed.3002042 21490276 PMC4927975

[27] Olagnier D, Lavergne RA, Meunier E, Lefèvre L, Dardenne C, Aubouy A, Benoit-Vical F, Ryffel B, Coste A, Berry A, Pipy B. Nrf2, a PPARγ alternative pathway to promote CD36 expression on inflammatory macrophages: implication for malaria. PLoS Pathog. 2011; 7:e1002254. 10.1371/journal.ppat.1002254 21949655 PMC3174257

[28] Jia K, Thomas C, Akbar M, Sun Q, Adams-Huet B, Gilpin C, Levine B. Autophagy genes protect against Salmonella typhimurium infection and mediate insulin signaling-regulated pathogen resistance. Proc Natl Acad Sci USA. 2009; 106:14564–69. 10.1073/pnas.0813319106 19667176 PMC2731839

[29] Wei Y, Kenyon C. Roles for ROS and hydrogen sulfide in the longevity response to germline loss in Caenorhabditis elegans. Proc Natl Acad Sci USA. 2016; 113:E2832–41. 10.1073/pnas.1524727113 27140632 PMC4878494

[30] Liu Y, Samuel BS, Breen PC, Ruvkun G. Caenorhabditis elegans pathways that surveil and defend mitochondria. Nature. 2014; 508:406–10. 10.1038/nature13204 24695221 PMC4102179

[31] Celli J, Tsolis RM. Bacteria, the endoplasmic reticulum and the unfolded protein response: friends or foes? Nat Rev Microbiol. 2015; 13:71–82. 10.1038/nrmicro3393 25534809 PMC4447104

[32] Yoneda T, Benedetti C, Urano F, Clark SG, Harding HP, Ron D. Compartment-specific perturbation of protein handling activates genes encoding mitochondrial chaperones. J Cell Sci. 2004; 117:4055–66. 10.1242/jcs.01275 15280428

[33] Shen X, Ellis RE, Lee K, Liu CY, Yang K, Solomon A, Yoshida H, Morimoto R, Kurnit DM, Mori K, Kaufman RJ. Complementary signaling pathways regulate the unfolded protein response and are required for C. elegans development. Cell. 2001; 107:893–903. 10.1016/S0092-8674(01)00612-2 11779465

[34] Choe KP, Leung CK, Miyamoto MM. Unique structure and regulation of the nematode detoxification gene regulator, SKN-1: implications to understanding and controlling drug resistance. Drug Metab Rev. 2012; 44:209–23. 10.3109/03602532.2012.684799 22656429 PMC3398467

[35] Al-Sawaf O, Clarner T, Fragoulis A, Kan YW, Pufe T, Streetz K, Wruck CJ. Nrf2 in health and disease: current and future clinical implications. Clin Sci (Lond). 2015; 129:989–99. 10.1042/CS20150436 26386022

[36] de Haan JB. Nrf2 activators as attractive therapeutics for diabetic nephropathy. Diabetes. 2011; 60:2683–84. 10.2337/db11-1072 22025774 PMC3198074

[37] Sporn MB, Liby KT. NRF2 and cancer: the good, the bad and the importance of context. Nat Rev Cancer. 2012; 12:564–71. 10.1038/nrc3278 22810811 PMC3836441

[38] Soares MP, Ribeiro AM. Nrf2 as a master regulator of tissue damage control and disease tolerance to infection. Biochem Soc Trans. 2015; 43:663–68. 10.1042/BST20150054 26551709 PMC4613525

[39] Suzuki T, Motohashi H, Yamamoto M. Toward clinical application of the Keap1-Nrf2 pathway. Trends Pharmacol Sci. 2013; 34:340–46. 10.1016/j.tips.2013.04.005 23664668

[40] Scialò F, Sriram A, Fernández-Ayala D, Gubina N, Lõhmus M, Nelson G, Logan A, Cooper HM, Navas P, Enríquez JA, Murphy MP, Sanz A. Mitochondrial ROS Produced via Reverse Electron Transport Extend Animal Lifespan. Cell Metab. 2016; 23:725–34. 10.1016/j.cmet.2016.03.009 27076081 PMC4835580

[41] Lavigne JP, Audibert S, Molinari N, O’Callaghan D, Keriel A. Influence of a high-glucose diet on the sensitivity of Caenorhabditis elegans towards Escherichia coli and Staphylococcus aureus strains. Microbes Infect. 2013; 15:540–49. 10.1016/j.micinf.2013.04.006 23639525

[42] Shore DE, Carr CE, Ruvkun G. Induction of cytoprotective pathways is central to the extension of lifespan conferred by multiple longevity pathways. PLoS Genet. 2012; 8:e1002792. 10.1371/journal.pgen.1002792 22829775 PMC3400582

[43] Kamath RS, Fraser AG, Dong Y, Poulin G, Durbin R, Gotta M, Kanapin A, Le Bot N, Moreno S, Sohrmann M, Welchman DP, Zipperlen P, Ahringer J. Systematic functional analysis of the Caenorhabditis elegans genome using RNAi. Nature. 2003; 421:231–37. 10.1038/nature01278 12529635

[44] Cai Y, Wei YH. Stress resistance and lifespan are increased in C. elegans but decreased in S. cerevisiae by mafr-1/maf1 deletion. Oncotarget. 2016; 7:10812–26. 10.18632/oncotarget.7769 26934328 PMC4905441

[45] Li X, Matilainen O, Jin C, Glover-Cutter KM, Holmberg CI, Blackwell TK. Specific SKN-1/Nrf stress responses to perturbations in translation elongation and proteasome activity. PLoS Genet. 2011; 7:e1002119. 10.1371/journal.pgen.1002119 21695230 PMC3111486

