# Supplementary material for: Glucose negatively affects Nrf2/SKN-1-mediated innate immunity in C. elegans
Source: Aging (Albany NY). 2018 Nov 15;10(11):3089–103. doi: 10.18632/aging.101610 (PMC6286829; doi:10.18632/aging.101610)
Supplement: Supplementary Figures [file aging-10-101610-s001.pdf]

SUPPLEMENTARY FIGURES

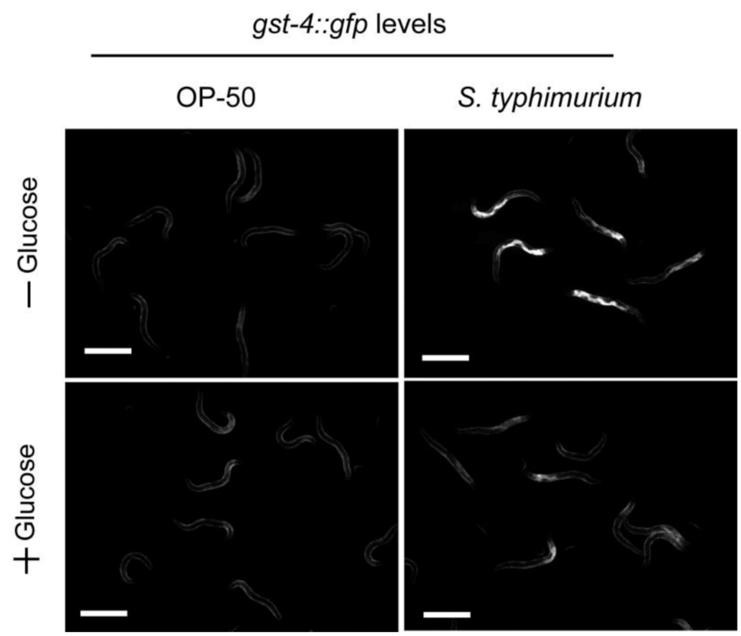

Figure S1. (Related to Figure 2A) Glucose reduces gst-GFP levels in response to *S. typhinurium* but not non-pathogenic OP-50 bacteria. *C. elegans* expressing the SKN-1 reporter *gst-4*::*gfp* were cultured on OP-50 bacteria in medium supplemented with and without 0.5% glucose from L1 stage to L4/young adult stage, then transferred to plates with either OP-50 bacteria plate or *S. typhimurium* bacteria for 2 days for infection. Images were then taken with Leica microscope under GFP channel. Scale bars are 600 µm.

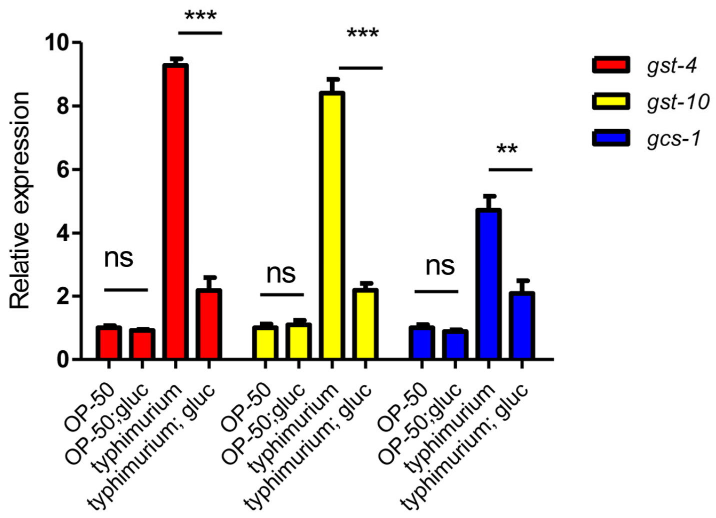

Figure S2. (Related to Figure 2C) glucose specifically affect S. typhimurium activation of SKN-1 target genes. Animals raised on medium with and without glucose from L1 to L4/young adult stage were infected with and without *S. typhimurium* for 2 days. mRNA were extracted and reverse transcribed to cDNA. Quantitative RT-PCR of SKN-1 target genes (*gst-4*, *gst-10*, *gcs-1*) was conducted using established primer sets and protocols. Shown are representative data from 1 of 2 independent experiments. Error bars indicate standard error of the mean (SEM) of 3 replicates. P values were obtained by student's t-test. **, P<0.001; ***, P<0.0001; ns, not significant.

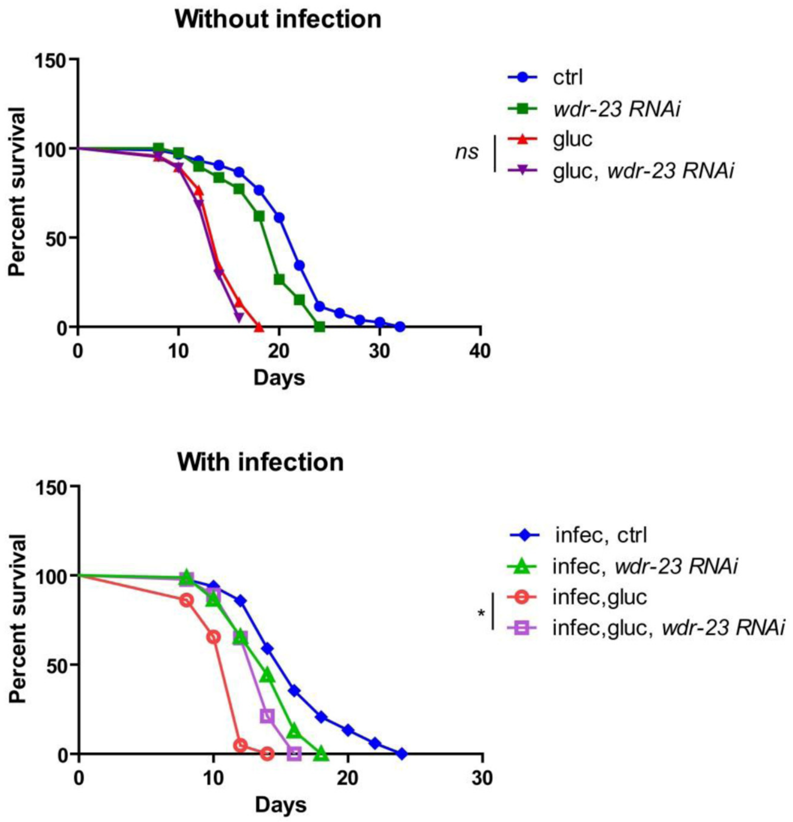

Figure S3. (Related to Figure 4D) wdr-23 RNAi cannot attenuate glucose's negative effect on lifespan of non-infected *C. elegans*. Lifespan and infection were carried out at 20 ºC. Animals fed bacteria expressing double-stranded RNA of *wdr-23* from L1 stage to L4/young adult stage on medium with and without glucose were infected with S. *typhimurium* for 2 days. Worms were then transferred to non-infected RNAi bacterial plates. Survival was recorded every other day until all worms died. number of worms >100 for each sample. See Table S5 for details. ns, not significant, * P<0.01.

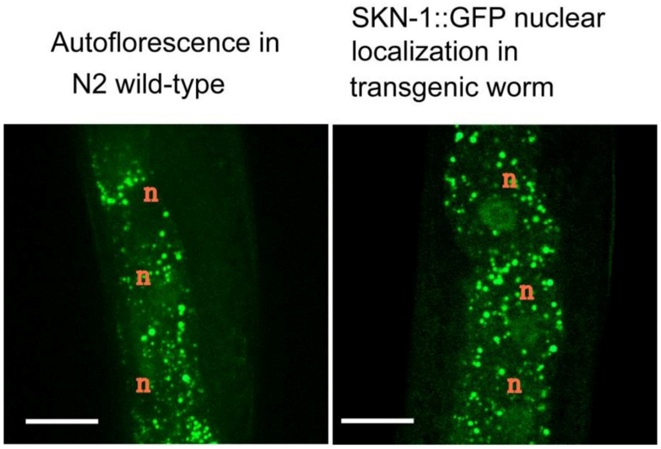

Figure S4. (Related to Figure 2D) The punctate signals are auto-florescence in the intestine of *C. elegans.* Animals without (left) and with SKN-1::GFP transgenes (right) were raised on medium with and without glucose from L1 stage to L4/young adult stag, then infected with S. *typhimurium* for 2 days before imaging. "n" marks above the nucleus of intestinal cells. Scale bars are 40 µm.
