# Supplementary material for: Glucose negatively affects Nrf2/SKN-1-mediated innate immunity in C. elegans
Source: Aging (Albany NY). 2018 Nov 15;10(11):3089–103. doi: 10.18632/aging.101610 (PMC6286829; doi:10.18632/aging.101610)
Supplement: Supplementary Tables [file aging-10-101610-s002.pdf]

SUPPLEMENTARY TABLES

Table S1 (related to Figure 1B and 1C).

	Treatment	Death(censored)	Mean lifespan	Standard Deviation	P value
Expr. 1					
	OP50	97(11)	20.7	3.9	<0.0001
	OP50+gluc	72(15)	11.7	2.5	
	typhimurium	88(9)	14.2	3.6	<0.0001
	typhimurium+gluc	74(11)	11.0	2.1	
Expr. 2					
	OP50	74(8)	19.4	4.0	<0.0001
	OP50+gluc	60(7)	10.5	1.7	
	typhimurium	72(7)	15.4	3.5	<0.0001
	typhimurium+gluc	68(15)	10.4	1.6	
Combined					
	OP50	171(19)	20.1	4.0	<0.0001
	OP50+gluc	132(22)	11.2	2.3	
	typhimurium	160(16)	14.7	3.6	<0.0001
	typhimurium+gluc	142(26)	10.7	1.9	

Table S2 (related to Figure 3C).

	Treatment	Death(censored)	Mean lifespan	Standard Deviation	P value
Expr. 1					
	Control	84(7)	15.0	4.0	
	skn-1 RNAi	79(11)	12.3	2.6	
	Glucose	69(6)	10.9	1.9	ns
	skn-1 RNAi +gluc	70(11)	10.5	1.8	
Expr. 2					
	Control	69(8)	15.8	4.1	
	skn-1 RNAi	75(10)	13.1	1.9	
	Glucose	56(16)	10.4	2.5	ns
	skn-1 RNAi +gluc	67(9)	11.1	2.0	
Combined					
	Control	153(15)	15.4	4.1	
	skn-1 RNAi	154(21)	12.7	1.9	
	Glucose	125(22)	10.6	2.6	ns
	skn-1 RNAi +gluc	137(20)	10.8	1.9	

Table S3 (related to Figure 4D).

	Treatment	Death(censored)	Mean lifespan	Standard Deviation	P value
Expr. 1					
	Control	84(7)	15.0	4.0	
	wdr-23 RNAi	76(6)	13.7	2.9	
	Glucose	69(6)	10.9	1.9	<0.0001
	wdr-23 RNAi +gluc	78(5)	13.7	3.4	
Expr. 2					
	Control	74(8)	16.3	3.8	
	skn-1 RNAi	78(4)	13.3	2.8	
	Glucose	65(14)	10.6	1.8	<0.0001
	wdr-23 RNAi +gluc	71(12)	12.5	2.7	
Combined					
	Control	158(15)	15.6	4.0	
	wdr-23 RNAi	154(10)	13.5	1.9	
	Glucose	134(20)	10.8	2.9	<0.0001
	wdr-23 RNAi +gluc	149(17)	13.1	3.2	

Table S4. Primers for quantitative real time PCR.

Gene name	Forward primer sequence (5'-3')	Reverse primer sequence (5'-3')	ref.
gst-4	CCCATTTTACAAGTCGATGG	CTTCCTCTGCAGTTTTTCCA	[1]
gst-10	GTCTACCACGTTTTGGATGC	ACTTTGTCGGCCTTTCTCTT	[1]
gcs-1	AATCGATTCCTTTGGAGACC	ATGTTTGCCTCGACAATGTT	[1]
skn-1	GTTCCCAACATCCAACTACG	TGGAGTCTGACCAGTGGATT	[1]
sek-1	TGCTCAACGAGCTAGACG	ATGTTCGACGGTTTCACG	[2]
pmk-1	CGACTCCACGAGAAGGAT	ATATGTACGACGGGCATG	[2]
gsk-3	GAGAAGAAGGATGAACTCTAC	TGTTGTCGCTGCTTCGAATA	
hsp-6	GGATGCTGGACAAATCTCTG	ACAGCGATGATCTTATCTCCA	[3]
hsp-60	CAAGGCTCCAGGATTCG	AAAGATCGTTGCTCCCG	[1]
hsp-3	ACCATCCAGGTCTTCGAAGG	AACCTCAATTTGTGGAACTCCG	[3]
hsp-4	CATCTCGTGGAATCAA CCCT	ACTTAGTCAT GACTCCTCCG	[3]
pek-1	GCCTCCCGTTGTTGGAAATA	CTGTCAGATCCTCCATGCAATC	
atf-6	AACTCGGTTCCCAAACTATCG	GTCCCTGTCACTTCACAATCA	
act-1	TCGGTATGGGACAGAAGGAC	CATCCCAGTTGGTGACGATA	[1]

Table S5 (related to Figure S3).

	Treatment	Death(censored)	Mean lifespan	Standard Deviation	P value
no infection	Control	79(14)	21.2	4.6	
	wdr-23 RNAi	79(12)	19.0	3.6	
	Glucose	62(30)	13.5	2.4	n.s.
	wdr-23 RNAi +gluc	66(22)	131	2.1	
infection					
	Control	70(16)	16.0	3.7	
	wdr-23 RNAi	65(23)	13.0	1.6	
	Glucose	83(4)	11.1	1.6	<0.01
	wdr-23 RNAi +gluc	73(9)	14.1	2.6	
